# Viral Interactions with PDZ Domain-Containing Proteins—An Oncogenic Trait?

**DOI:** 10.3390/pathogens5010008

**Published:** 2016-01-18

**Authors:** Claire D. James, Sally Roberts

**Affiliations:** 1 Institute of Cancer and Genomic Sciences, College of Medical and Dental Sciences, University of Birmingham, Vincent Drive, Birmingham B15 2TT, UK; cdjames@vcu.edu; 2 Present address; Virginia Commonwealth University, School of Dentistry, W. Baxter Perkinson Jr. Building, 521 North 11th Street, P.O. Box 980566, Richmond, VA 23298-0566, USA

**Keywords:** oncogenic viruses, PDZ proteins, cell polarity, PI3K/AKT signalling, HPV E6, adenovirus E4ORF1, DLG1, SCRIB, tight junctions

## Abstract

Many of the human viruses with oncogenic capabilities, either in their natural host or in experimental systems (hepatitis B and C, human T cell leukaemia virus type 1, Kaposi sarcoma herpesvirus, human immunodeficiency virus, high-risk human papillomaviruses and adenovirus type 9), encode in their limited genome the ability to target cellular proteins containing PSD95/ DLG/ZO-1 (PDZ) interaction modules. In many cases (but not always), the viruses have evolved to bind the PDZ domains using the same short linear peptide motifs found in host protein-PDZ interactions, and in some cases regulate the interactions in a similar fashion by phosphorylation. What is striking is that the diverse viruses target a common subset of PDZ proteins that are intimately involved in controlling cell polarity and the structure and function of intercellular junctions, including tight junctions. Cell polarity is fundamental to the control of cell proliferation and cell survival and disruption of polarity and the signal transduction pathways involved is a key event in tumourigenesis. This review focuses on the oncogenic viruses and the role of targeting PDZ proteins in the virus life cycle and the contribution of virus-PDZ protein interactions to virus-mediated oncogenesis. We highlight how many of the viral associations with PDZ proteins lead to deregulation of PI3K/AKT signalling, benefitting virus replication but as a consequence also contributing to oncogenesis.

## 1. Introduction

PSD95/DLG/ZO-1 (PDZ) domains are one of the most widely distributed protein-protein interaction domains; the human genome encodes over 320 PDZ domain-containing proteins [1]. The domain itself is an evolutionarily conserved domain of approximately 90 amino acids folded into six β-sheets (βA–βF) and two α-helices (αA–αB), named for its presence in PSD95, DLG, and ZO-1 proteins. The domains are often found in combination with a number of other protein-protein interaction modules, for example members of the membrane-associated guanylate kinase (MAGUK) family include an SH3 and inactive guanylate kinase domain, as well as multiple PDZ modules. The presence of modular domains within a single protein allows PDZ domain-containing proteins (herein referred to as PDZ proteins) to form interactions with many partner proteins simultaneously and act as a scaffold; thus these proteins are involved in myriad of functions, including organizing multiprotein signalling complexes at the immunological and neurological synapses, forming cell-cell junctions, cell proliferation and survival, intracellular trafficking, apico-basal and planar polarity. Perhaps not surprisingly considering the wide remit of PDZ protein functions, deregulation of a number of PDZ proteins has been linked to tumourigenesis [2]. However, it is often not clear whether to define some PDZ proteins as oncogenes or tumour suppressors, as their roles in tumourigenesis is context-dependent [3,4]. Whilst abundance and/or cellular localization is a contributing factor in PDZ protein function, tissue specificity is also of importance; this is the case for the epithelial polarity protein SCRIB, which has a tumour suppressor role in epithelia but a pro-oncogenic role in myc-driven lymphomas [5,6].

Binding to the PDZ domain is mediated by short peptide sequences—PDZ-binding motifs (PBM)—often but not always found at the carboxy terminus of proteins. PDZ domains are grouped into specificity classes based on the consensus sequence of the PBM recognized. Early studies defined three main classes of specificity; class I S/T-X-φ-COOH, class II φ- X-φ-COOH and class III D/E-X-φ-COOH, where φ is an hydrophobic residue, but more recent analyses using protein arrays and peptide libraries have defined at least 16 distinct specificity classes (the reader should refer to the references for details of the consensus sequences) [1,7]. In binding, the PBM sits into the hydrophobic groove of the PDZ domain formed between one of the β sheets (βB) and the α helix αB, with a highly conserved carboxylate binding loop at the base. Amino acids outside the PBM contribute to PDZ substrate specificity of binding, and phosphorylation of the PBM can act as a negative or positive regulator of PDZ binding [1,7]. Likewise, phosphorylation within the PDZ domain itself is also a regulatory mechanism of ligand binding [1].

A number of diverse but highly pathogenic viruses encode proteins that target PDZ domain containing proteins, often but not always, using a PBM. Pathogenicity of both the SARS coronavirus (SARS-CoV), a positive strand RNA virus that causes severe acute respiratory infections, and the neurotropic rabies virus, are linked to PDZ-binding functions of their envelope proteins. Recombinant SARS-CoV expressing the envelope E protein lacking the C-terminal PBM had no effect on viral growth in a murine infection model, but caused less lung damage and mortality than in mice infected with the wild type virus [8,9]. The E protein PBM binds to the tight junction associated PDZ protein PALS1, leading to disturbed tight junction formation of polarized epithelia. It can also induce proinflammatory cytokine expression, possibly through PBM binding of the PDZ protein syntenin-1. Both of these PBM functions could be determinants of the lung pathobiology associated with SARS-CoV infection [8,10]. Survival of rabies infected neuronal cells is associated with the ability of the viral envelope G protein to interact with the PDZ domain-containing serine threonine kinase MAST2, leading to the disruption of the MAST2-PTEN complex that is intimately involved in the inhibition of neuronal survival [11]. In an attenuated strain, a mutation within the G protein PBM confers an expansion of the PDZ proteins bound, and leads to apoptosis of neuronal cells [12]. Also, the canonical class I PBM at the C-terminus of the NS1 protein of over 99% type A influenza viruses is a good target for attenuation of these pathogenic viruses [13,14,15,16]. Removal of the PBM decreases efficacy of influenza H1N1 virus transmission, as well as virus replication, and increases interferon expression in infected cells [17,18]. Moreover, switching the H1N1 PBM for the sequence of an avian-specific strain increased virus virulence [19]. The NS1 PBM of the highly pathogenic strain H5N1 interacts with the PDZ domains of several PDZ proteins, including the epithelial polarity regulators DLG1 and SCRIB and the tight junction protein MAGI-1 [20,21,22]. Using models of influenza A infection, this motif has been shown to contribute to the pathogenesis of these viruses by disruption of tight junction integrity of polarized epithelia, also disruption of cell junctions may benefit viral dissemination within the host, and/or spread to other hosts [21]. Furthermore, relocalization of SCRIB to cytoplasmic puncta in infected cells is associated with the abrogation of the polarity protein’s pro-apoptotic function and may therefore protect infected cells from viral induction of apoptotic pathways, thus supporting viral propagation [20,23].

**Figure 1:**
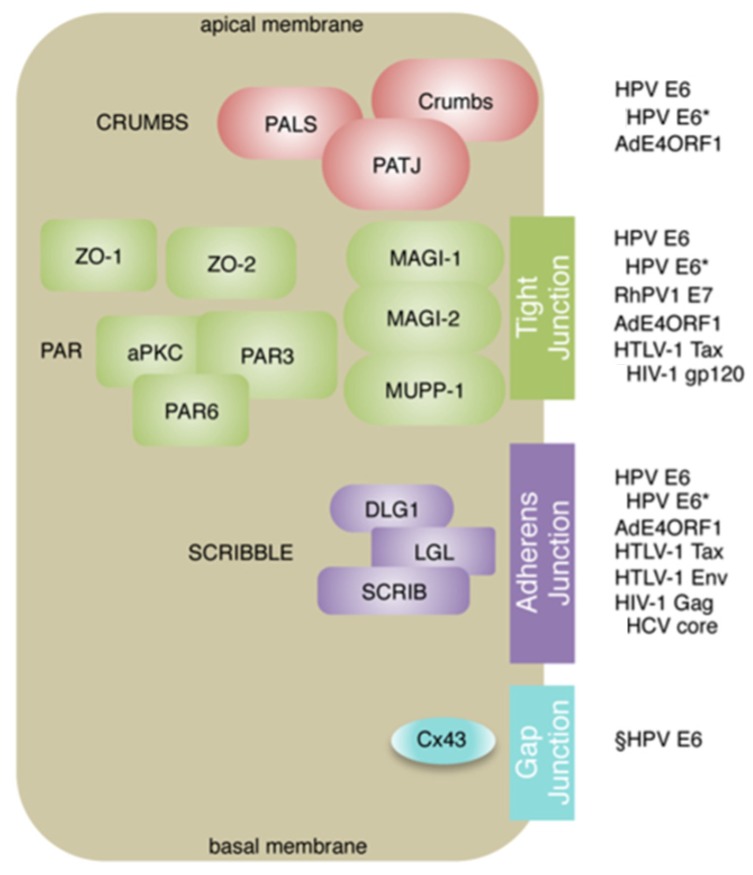
Oncogenic viruses target a common subset of PDZ proteins. Cellular proteins targeted by the viruses are often components of the polarity complexes Crumbs, Par and Scribble that regulate apico-basal polarity and the formation of intercellular junctions including tight, adherens and gap junctions. The virus proteins that target components of the different complexes are given. ¶These viral proteins do not target the PDZ proteins using a classical PBM. §HPV E6 forms a complex with DLG1 and Cx43 to disturb trafficking. Adapted from Javier R. T., Rice, A. P. 2011 [55].

Targeting PDZ protein function is also a common function amongst viruses classified as human carcinogens by the World Health Organization [24]. As well as having roles in virus infectivity, the virus-PDZ protein associations are important in virus entry, replication and for some of these viruses, cell transformation. These include the hepatitis viruses B and C, Kaposi sarcoma herpesvirus, human T cell leukaemia virus type 1, high-risk human papillomaviruses, and human immunodeficiency virus type 1. In this review, we examine the interactions between these cancer-causing viruses and PDZ proteins and discuss their contribution to pathogenesis of this important group of viruses. Whilst HIV-1 is defined as a group I carcinogen, it contributes indirectly to carcinogenesis via immunosuppression and infected individuals have higher incidence rates of tumours driven by other cancer-causing viruses, and so it is also included in the review. Interactions with PDZ proteins are also pivotal in the formation of mammary tumours in mice infected by the human adenovirus type 9 and although this virus is not linked to human cancers, the study of Ad9 interactions with PDZ proteins has given valuable insight in viral carcinogenesis. The different virus-PDZ associations are listed in Table 1 and common PDZ targets are shown in Figure 1.

**Table 1:** Virus interactions with PSD95/DLG/ZO-1 (PDZ) proteins ^1^.

Virus/Viral Protein	PDZ Target	PBM Mediated Interaction (Type, Sequence)	Virus Mediated Effect upon PDZ Target (If Known)	References
HCV Core protein	DLG1, SCRIB	No	Decreased expression (DLG1); mislocalization (SCRIB)	[25]
HBV Core protein	GIPC1 (also known as TIP-2), PTPN3	Yes (non-typical, SQC)	Disruption of GIPC1 interaction with LPA signalling molecule (?)	[26,27]
KSHV	PDLIM2	No	Suppresses PDLIM2 transcription by increased promoter methylation	[28]
HIV-1 Gag	PDZD8, DLG1	No	Stabilizes viral capsid (PDZD8 [?]); colocalization in plasma membrane, antagonistic to virus infectivity (DLG1)	[29,30]
HIV-1 Env	SDCBP1 (Syntenin-1)	No	Recruited to the plasma membrane during virus attachment, antagonistic to virus infectivity (?)	[31]
HIV-1 Glycoprotein 120	ZO-1	No	Displacement from tight junctions, reduced ZO-1 transcription	[32]
HTLV-1 Env	DLG1	Yes (class I, SSL)	Colocalization in plasma membrane, at possible virological synapse, also colocalization with major HTLV-1 receptor GLUT-1	[33]
HTLV-1 Tax	DLG1, SCRIB, MAGI-1, MAGI-3, Pro-IL-16, TIP-1	Yes (class I, TEV)	Altered subcellular localization (DLG1, SCRIB, MAGI-1)	[34,35,36,37]
Ad E4ORF1	DLG1, MAGI-1, MUPP1,PATJ, ZO-2	Yes (class I, TLV)	Altered subcellular localization (MAGI-1, MUPP1, PATJ, ZO-2), associated with tight junction disruption, accumulate at plasma membrane (DLG1) where activates PI3K signalling	[38,39,40,41,42]
Beta-HPV8 E6	SDCBP2 (Syntenin-2)	No	Repression of SDCBP2 transcription	[43]
RhPV1 (MmPV1) E7	PARD3 (PAR3)	Yes (class I, SRV)	Degradation via the proteasome	[44]
High-risk alpha-HPV E6	Includes DLG1, SCRIB, MAGI-1, MAGI-2, MAGI-3, PTPN3, PTPN13, PATJ, PAR3, SDCBP2 (see Table 2 for full list)	Yes (class I, S/TXV/L)	Degradation via the proteasome, transcriptional downregulation, altered subcellular localization, complex formation to alter function	See Table 2
^2^High-risk alpha-HPV E6*	DLG1, MAGI-1, PATJ, SCRIB	No	Degradation via the proteasome	[45,46]

^1^ Interactions between virus proteins and PDZ domains/proteins have also been identified from peptide/protein screens but they are not included in this table [47,48,49]; ^2^ HPV E6* protein is an E6 isoform that lacks the PDZ-binding motifs (PBM). (?) Function/effect not proven.

## 2. Oncogenic Viruses and PDZ Proteins

### 2.1. Hepatitis C virus (HCV)

**Figure 2:**
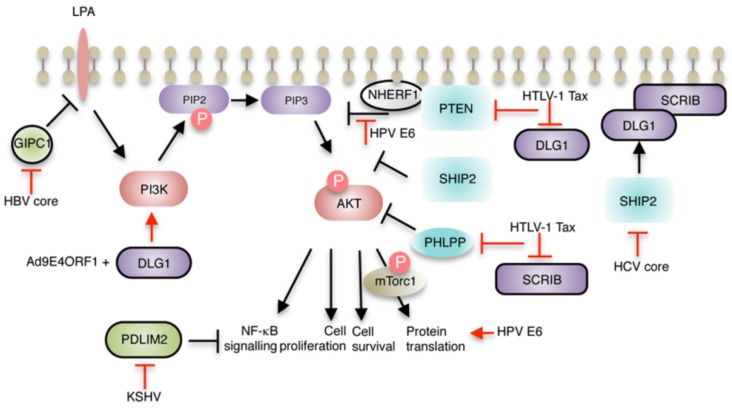
The effects of virus mediated targeting of PDZ proteins often converge on the PI3K/AKT signal transduction pathway. A schematic of the PI3K/AKT pathway showing the PDZ proteins involved that are targets of the viral proteins. PDZ proteins are identified by black outline. PIP2 and PIP3 are phosphoinositides. The reader should refer to the text for a fuller explanation of the effects of the virus proteins on the signalling pathway.

A common risk factor for hepatocellular carcinoma is chronic infection with HCV. The virus primarily infects the highly polarized epithelium in the liver and depolarization of this tissue by the virus is critical for virus entry and cell-to-cell transmission of virus. Entry of HCV occurs at tight junctions and most likely requires disruption of these structures, contributing to depolarization of the tissue [50]. The tight junction protein claudin 1 and the major receptor for high-density lipoproteins, SR-B1 (class B scavenger receptor) are important entry factors for HCV and both proteins encode PBM at the C-termini. The interaction between SR-B1 and a PDZ domain-containing adaptor partner PDZK1 facilitates virus entry [51]. Expression of the HCV core protein disrupts the apicobasal polarity of polarized epithelia and leads to deregulation of components of the Scribble polarity complex DLG1 and SCRIB at cell-cell contacts. DLG1 protein expression is decreased and there is mislocalisation of SCRIB from cell-cell contacts in liver epithelial cells [25]. The core protein does not target the polarity PDZ proteins directly, but subverts the phosphoinositide (PI) phosphastase SHIP2, leading to a decrease in production of the PI phosphatidylinositol 4,5 bisphosphate (PIP2) which has a role in the targeting of DLG1 and SCRIB to the basolateral membrane (Figure 2). Such deregulation of the polarity pathways regulated by the Scribble components may be a contributing factor in HCV driven oncogenesis [25]. Also, it has been reported that the carboxyl sequence of the poorly characterized non-structural protein NS4B encodes a class I PBM-like sequence (X-T-X-C); however, as yet there is no experimental evidence that supports interaction between NS4B and PDZ proteins [52]. Nevertheless, propagation of other RNA viruses within Flaviviridae (tick borne encephalitis virus, West-Nile virus and dengue virus) is dependent upon PBMs within the non-structural NS5 proteins, and have been shown to bind to various PDZ proteins, including those involved in polarity control and tight junction assembly and maintenance; and mutations in these PBM negatively affect viral replication [53,54].

### 2.2. Hepatitis B virus (HBV)

HBV is another virus whose infection of the liver causes acute and chronic hepatitis with an increased risk of the development of hepatocellular carcinoma. Like the HCV core, the activities of the core protein of HBV (HBVc) are linked to viral pathogenicity. Yeast two hybrid screening for HBV core partners identified the PDZ protein GIPC1 (GAIP-interaction protein, C-terminus, family member 1, also known as TIP-2) as a robust binding partner. The interaction is mediated by the core’s PBM, which has sequence similarities with other ligands that bind GIPC1 (X-X-C-COOH, a non-typical PBM [56]), and deletion of the carboxyl cysteine from the HBVc was sufficient to block the interaction [26]. The consequences of this HBVc activity in HBV pathogenesis is unknown, but as GIPC1 is a scaffold protein important in the endocytic trafficking of multiple signal transduction receptors, the association may have an important role in facilitating movement of the HBV capsids to the nucleus [57]. It is possible that the association between the core and GIPC1 is linked to the virus’s role in carcinogenesis via disruption of interactions of cellular substrates with the PDZ domain of GIPC1. Pertinent to this is the interaction between lysophosphatidic acid (LPA) and the PDZ domain of GIPC1, which is necessary to attenuate LPA signalling (Figure 2). Loss of this control leads to increased cell proliferation and survival through the resulting upregulation of the AKT pathway [57]. This activity may function synergistically with the virus’s HBx activation of AKT signalling to block apoptosis of infected hepatocytes, thus enabling persistence of this viral carcinogen [58].

The HBVc can also bind via its PBM to the non-receptor protein tyrosine phosphatase PTPN3; however the precise role of this association in HBV pathogenesis is not known. PTPN3 has been shown to have a suppressive role on HBV gene expression, but this control is independent of the PBM activity of the HBVc [27].

### 2.3. Kaposi Sarcoma Herpesvirus (KSHV)

KSHV (also known as human herpesvirus 8) is the aetiological agent of Kaposi sarcoma, primary effusion lymphoma and multicentric Castleman disease and KSHV induced cancers are particularly common among patients with acquired immunodeficiency syndrome (AIDS). The route to carcinogenesis is not fully understood, but as with all cancer-causing viruses it involves dysregulation of signalling pathways important for host proliferation and survival. Notably, a critical mechanism is the constitutive activation of the nuclear factor κB (NF-κB) and signal transducer and activator of transcription 3 (STAT3) signalling pathways. In normal signalling, the tumour suppressor PDZ-LIM domain-containing protein (PDLIM2, the LIM domain is a protein:protein interaction domain composed of two contiguous zinc fingers) regulates turnover of RelA (p65), one of the components of the NF-κB, and STAT3 by ubiquitination and degradation via the proteasome [59]. This normal mechanism of turnover of STAT3 and NF-κB is deregulated in KSHV pathogenesis by the virus repressing PDLIM2 transcription by increased DNA methylation of the promoter region [28].

### 2.4. Human Immunodeficiency Virus Type 1 (HIV-1)

HIV-1 is a lentivirus that infects several different types of immune cells, but primarily CD4^+^ positive T cells, and causes AIDS, a condition that is characterized by immune suppression, and AIDS patients are at high-risk of opportunistic microbial infections, including cancer-causing viruses. Interactions between HIV-1 and host PDZ proteins have been reported to take place at early and late stages of virus infection. The HIV-1 Gag polyprotein interacts with PDZ proteins, although the interactions are not mediated by a classical PBM. The cytoskeletal protein PDZD8 interacts with Gag involving those regions of the polyprotein associated with early post-entry events; knockdown of this cellular cofactor inhibits HIV-1 infection and negatively affects viral capsid stability, whereas PDZD8 overexpression enhances infection by HIV-1 [29,60]. PDZD8 is a poorly understood protein that has been shown to regulate microtubule stability through an interaction with moesin and microtubule stability at the viral synapse is likely to be important for HIV-1 infection [61,62]. However, recent findings using cell lines in which PDZD8 expression has been eliminated by the CRISPR-CAS system indicated that HIV-1 infection is not affected by loss of this host protein [63]. The other known PDZ target of Gag is DLG1 [30]. Binding to DLG1 is mediated through the nucleocapsid domain of Gag and the viral protein associates with an isoform of DLG1 that preferentially locates to the plasma membrane, where the two proteins colocalize [30]. The Gag—DLG1 association regulates HIV-infectivity, such that depletion of DLG1 enhanced infectivity and DLG1 overexpression reduced particle infectivity [30]. Loss of DLG1 from infected cells leads to the redistribution of Env and Gag from predominantly plasma membrane sites to intracellular vesicle-like structures that function as specialized platforms for the generation of infectious particles and suggests that DLG1 regulates plasma membrane dynamics in HIV-1 infected T cells [30]. DLG1 is not the only PDZ protein to act antagonistically in HIV-1 infectivity; syntenin-1 is recruited to the plasma membrane during HIV-1 attachment by the viral envelope glycoprotein (Env) and associates with the main HIV-1 receptor CD4 [31]. Over expression of syntenin-1 inhibits HIV production and silencing increases viral entry, most likely by inducing actin polymerization and accumulation at the viral attachment site to inhibit virus entry [31].

HIV-1 transmission occurs at mucosal epithelial surfaces and one of the consequences of HIV-1 infection is increased permeability of the intestinal tract leading to an enhancement in microbial infections. In polarized epithelial cell cultures, HIV-1 infection causes a displacement of the PDZ protein ZO-1 from the tight junctions in the apical region of the membrane, followed by a marked reduction in the transcription of ZO-1 and other tight junction proteins [32]. The disruption of tight junction integrity is mediated by the HIV-1 glycoprotein 120, which induces upregulation of the inflammatory cytokine TNF-α, a known disruptor of tight junctions [32,64].

### 2.5. Human T Cell Leukaemia Virus (HTLV-1)

HTLV-1 is a retrovirus that is the aetiological agent of the highly aggressive adult T cell leukaemia and primarily replicates in CD4^+^ T cells. Transmission of the virus occurs predominantly by direct contact between infected and target cells; the site of contact forms a virological synapse where the Gag glycoprotein and envelope (Env) protein, together with the viral RNA become concentrated. The Env protein contains at its C-terminus a class I PBM and the activity of this motif contributes to the ability of Env to trigger cell-to-cell fusion as judged by analysis of syncytium formation—a marker of cell-to-cell fusion [33]. There is also evidence that the PBM regulates Env trafficking since disruption of the motif causes accelerated endocytosis and degradation of the Env protein [65]. Thus, binding to PDZ proteins may be important for the proper localization of Env—directing it to specific microdomains in the plasma membrane. To date, only one PDZ protein, DLG1 has been identified as binding to Env in a PBM dependent manner. Env and DLG1 colocalize at sites characteristic of virological synapses on HTLV-1-infected T cells [33]. Moreover, depletion of DLG1 reduced syncytium formation, although Env expression was not affected suggesting that additional PDZ proteins might be involved in regulating Env trafficking [65,66]. Since there is a partial colocalization between DLG1 and the major HTLV-1 receptor GLUT-1 (glucose transporter type 1) at intercellular contacts formed between infected and target cells, a possible function for this interaction may be to aid efficient clustering of GLUT-1 at the polarised synapse [33].

The HTLV-1 Tax transcription factor also contains a canonical class I PBM at its C–terminus. Tax is the major oncogenic determinant of HTLV-1; when expressed alone it induces the transformation of rat fibroblasts in culture and is capable of promoting leukaemia in transgenic mice [67,68,69]. Studies of the contribution of the HTLV-1 motif to the transformation functions of Tax have shown that deletion of the motif reduces the transformation potential of Tax in rat fibroblasts [67]. The domain is also necessary for T cell overproliferation and reconstruction of a Tax PBM deletion mutation in the context of whole HTLV-1 virus showed that the motif was critical for induction of proliferation of human lymphocytes following viral infection [70,71]. Using this same model of HTLV-1 pathogenesis, the mutant Tax PBM deletion virus, whilst not necessary for HTLV-1 driven immortalization, was shown to be a requirement for acquisition of host genomic instability as judged by micronuclei formation, and the mutant virus was severely compromised in its ability to establish persistent infection in rabbits [70]. Taken together, the function of the Tax PBM plays a significant role in those characteristics of the virus necessary to induce T cell transformation. In further support of the role of the PBM in HTLV-1 driven transformation, a PBM is absent from the Tax protein of HTLV-2, a virus that is not leukemogenic. Interestingly, as for cellular PBM-PDZ interactions, the function of the Tax PBM is susceptible to regulation by phosphorylation [1]. The protein kinase casein kinase 2 phosphorylates the threonine residue within the Tax PBM and negatively regulates binding to PDZ proteins, suggesting that timing of PDZ targeting may be important in the HTLV-1 replication cycle and host transformation [72].

But what are the physiological targets of this domain? To date, Scribble polarity complex components DLG1 and SCRIB have been identified as robust binding partners of the Tax PBM and both polarity proteins have been shown to have critical roles in T cell signalling, including the formation and organization of the immunological synapse [34,35,71,73,74,75,76,77,78]. The subcellular localization of both proteins is affected by Tax expression; in HTLV-1 infected T cells, and in cells overexpressing Tax, DLG1 and SCRIB are relocated from cell membranes to cytoplasmic puncti or granules and this is concomitant with an increase in the insolubility of the polarity proteins, effects that are dependent on an intact Tax PBM [67,71,75,79]. The relocalization of DLG1 and SCRIB is a likely mechanism of inactivation of their normal function. Indeed, the ability of DLG1 to block cell cycle progression of fibroblasts is inhibited by Tax overexpression and is PBM dependent [35]. The inhibition of cell cycle progression is associated with an increase in phosphorylation of DLG1 and it is well known that cell cycle mediated phosphorylation of DLG1 regulates DLG1 localization and function [35,80]. So, is DLG1 inactivation necessary for T cell transformation by Tax? Whilst silencing expression of DLG1 in Tax expressing T cells augments both growth and transformation of a mouse T cell line, depletion of DLG1 does not complement transformation in the absence of the Tax PBM, altogether suggesting that HTLV-1 transformation does not depend solely on Tax binding and inactivating DLG1 [81].

Correct localization of DLG1 and SCRIB is important for the formation of signalling complexes at specific sites in the cell and Tax PBM mediated effects on these proteins is likely to lead to a disruption of these signalling pathways. Two critical signal transduction pathways for T cell signalling are the T-cell receptor (TCR)-induced activation *via* the transcription factor nuclear factor of activated T cells (NFAT), and the AKT pathway. In T cells, DLG1 is required for p38-mediated activation of NFAT and stabilization of PTEN to inhibit AKT-mediated signalling (Figure 2) [71,82]. Moreover, SCRIB may also organize receptor signalling through the NFAT pathway since overexpression of SCRIB attenuates NFAT activity and in Tax expressing T-cells, this activity of SCRIB was inhibited, whereas SCRIB orchestrated inhibition of NFAT still occurred when the Tax PBM was mutated [75]. In addition, in immortalized T cells, Tax stimulates AKT activating phosphorylation in a PBM dependent manner by a mechanism that involves decreased membrane localization of the phosphatases PTEN and PHLPP (PH domain and leucine rich repeat protein), a critical requisite for both phosphatases to negatively regulate AKT activity. In both cases, overexpression of membrane forms of the phosphatases overcame the effects of Tax on AKT phosphorylation. Tax-mediated mislocalization of these phosphatases from the plasma membrane may involve Tax disruption of DLG1 and SCRIB scaffolding functions; SCRIB is necessary for membrane localization of PHLPP [83] and PTEN is a binding partner of DLG1 [84]. How DLG regulates PTEN activity is not altogether clear, but DLG1 increases PTEN stability and augments PTEN’s inhibition function of the AKT pathway [85]. Interestingly, Tax attenuates binding between PTEN and DLG1 in T cells and this requires the activity of the PBM [78].

The HTLV-1 Tax protein has been shown to bind to many other PDZ proteins besides DLG1 and SCRIB (Table 1), but as yet the physiological relevance of these interactions to HTLV-1 infection and transformation is unknown. Singling out one of these, the T-cell specific interleukin-16 precursor (Pro-IL-16) binds to Tax via the PBM in human T-cells, where both proteins colocalize in the nucleus [86]. Pro-IL-16 is highly expressed in all T cells and is the precursor to IL-16. The presence of an intact PBM enables Tax-expressing cells to overcome pro-IL-16 driven cell cycle arrest, suggesting a role for the interaction in overcoming the growth inhibitory activity of pro-IL-16 and promoting cell cycle progression [86]. Such progression is favourable for viral propagation and is likely to contribute to the overproliferative phenotype of HTLV-1 transformed cells.

### 2.6. Adenoviruses (Ad)

Whilst not human carcinogens, Ad are potent carcinogens in rodent cells and are provocative agents for the study of carcinogenesis. This review will focus on Ad type 9 (Ad9); a human virus commonly associated with benign eye infections, but causative of mammary tumours in rats in the presence of high levels of the hormone oestrogen [87]. Unlike other human Ad which drive carcinogenesis by the actions of the products of the E1A and E1B genes, Ad9’s oncogenicity is determined by the E4 region ORF1 gene (E4ORF1) function alone [88]. Three regions of E4ORF1 are important in cell transformation, including a C-terminal class I PBM, which is known to bind to a number of PDZ domain-containing proteins, including DLG1 and tight junction proteins, MAGI-1, MUPP1, PATJ and ZO-2 [38,39,40,41] (Table 1). In fact, in polarized epithelial cells, the expression of E4ORF1 blocks localization of the tight junction proteins to the intercellular junctions and many of the proteins become sequestered to E4ORF1-containing structures in the cytoplasm. The disruption of tight junction function and the associated polarity defects are entirely dependent on an intact viral PBM [41]. In contrast to the effects upon the tight junction proteins, DLG1 is not sequestered into the cytoplasm but driven to accumulate at the plasma membrane.

A second intriguing function of the E4ORF1 PBM is the activation of PI3K pathway at the plasma membrane, a function that is dependent on the E4ORF1 PBM localizing the viral protein to sites in the membrane and triggering activation of downstream targets, including AKT/PKB [89]. Importantly, overexpression of individual PDZ targets of E4ORF1, including DLG1, suppressed E4-ORF1 mediated AKT stimulation, strongly suggesting a link between this activity and PDZ targeting. Since this function of the E4ORF1 PBM was not required for tight junction disruption, this represents a distinct activity mediated largely (but not entirely [90]) by the PBM. All together, these studies suggest that the different E4ORF1 functions—disruption of tight junctions and apicobasal polarity, and activation of the PI3K signalling pathway—cooperate to transform mammary cells.

The next part in this story went on to understand the mechanism of PI3K activation, and this led to a series of very elegant studies from the Javier laboratory. An important finding was that the increase in PI3K activity was not a consequence of inactivation of DLG1 function as first thought, but that E4ORF1 exerts a gain-of-function upon DLG1, by binding DLG1 and promoting a conformational change allowing binding to the plasma membrane to direct PI3K activation [42]. This led to the idea that DLG1, whilst widely regarded as a tumour suppressor can acquire oncogenic characteristics in a different cellular context [3]. The summation of these studies has shown that E4ORF1 forms complexes with PI3K and DLG1 in the cytoplasm; complex formation leads to increased stabilization of PI3K and increasing PI3K catalytic activity and activation of AKT. The complex is then recruited to the plasma membrane through the induction of changes to DLG1 conformation [91]. Deregulated activation of AKT thereby promoting cellular survival and metabolism, which are important for virus replication, but also necessary for adenovirus-mediated transformation (Figure 2). Indeed, the formation of the E4ORF1/PI3K/DLG1 ternary complex was necessary for anchorage independent cell growth—a robust indicator of cell transformation. In addition, the complex of E4ORF1 and DLG1 also deregulates epidermal growth factor receptor signalling leading to activation of nuclear MYC; an action thought to aid virion production in infected cells, although this action is not solely driven by the function of the E4ORF1 PBM [92].

The ability of E4ORF1 to activate PI3K in a DLG1 dependent fashion was found to be a conserved function between human Ad subgroups A through to D, including those members used as E4ORF1-encoding vectors in human gene therapy and vaccination, raising possible safety concerns of their use in humans [93].

### 2.7. High-Risk Human Papillomaviruses (HPV)

Whilst some 200 HPV types have been identified, only a small subset (25 types) of these are defined as carcinogens, including some as possible or probable carcinogens [24]. These viruses—referred to as high-risk types—infect the mucosal surfaces of the anogenital and oropharyngeal tracts and are associated with cancers at these sites (cervix, vulva, vagina, penile, anal, tonsil and base of tongue). HPV types 16 and 18 are the most common high-risk types, present in ~70% of cervical cancers which has a nearly 100% association with high-risk HPV infection, and HPV16 is found in over 90% of HPV driven oropharyngeal carcinomas. The E6 and E7 early viral proteins are the main drivers of oncogenesis and all express an E6 protein that contains a class I PBM at the C-terminus of the protein. The conservation of this motif amongst the high-risk viruses is a strong indication that a PDZ targeting function is important in their life cycle and as an oncogenic signature. Intriguingly, a recent analysis of the ability of different HPV types to target PDZ proteins has revealed that some of the non-cancer causing types can degrade PDZ proteins (e.g., HPV40 can degrade MAGI-1), suggesting that the ability to degrade PDZ proteins was acquired prior to the oncogeneic trait and that the ancestor of both high-risk and low-risk types acquired this new trait [94]. The authors of this study propose a model in which this new trait allowed these viruses to colonize new cellular niches (e.g., the cervical transformation zone), but for viral fitness and survival adapted to this site by acquiring additional functions such as hTert activation and p53 degradation, functions that are necessary for cell transformation [94].

Several systems have been used to demonstrate the importance of this E6 domain to HPV pathogenesis. Life cycle studies based on human keratinocytes transfected with whole HPV genomes in which the E6 PBM has been deleted have shown that PBM function is critical for the vegetative phase of the life cycle, with the mutant genome unable to support viral genome amplification or expression of the viral late proteins [95]. A role for the E6 PBM in viral episome maintenance was also identified in the life cycle models [95,96,97]. The various life cycle defects correlated with a marked reduction in proliferation of the mutant genome-containing cells, indicating that the E6 PBM regulates proliferation of the viral genome-containing and that this is important for multiple phases of HPV DNA replication [95,96]. Interestingly, although E6 also mediates the degradation of the tumour suppressor p53 in infected cells, further depletion of p53 protein by using p53-targetted siRNAs, or by the introduction of a dominant negative 53, into cells harbouring E6-PBM mutant HPV16 genome-containing cells, rescues maintenance of the viral genomes, suggesting that there is mutual cooperation between these two E6 functions in viral genome maintenance during the virus life cycle [98].

In agreement with the life cycle studies, expression of E6 in mammalian keratinocytes showed that the E6 PBM promotes cell proliferation, but also the acquisition of phenotypes linked to invasive and metastatic growth of tumours (epithelial mesenchymal transition and anchorage independent cell growth) [99,100,101,102,103]. In addition, the motif is necessary for the morphological transformation and induction of tumourigenesis of rodent cell lines and contributes to tumour development in a transgenic mouse model of cervical carcinogenesis [104,105]. However, the motif was not required for the immortalization of keratinocytes suggesting that the E6 PBM function is of more importance in the later stages of HPV driven carcinogenesis [106]. The link between this HPV function and cell transformation is further supported by the discovery that the E7 oncoprotein of the rhesus papillomavirus type 1 virus (RhPV1, also known as *Macaca mulatta* PV1 [MmPV1]), which drives mucosal neoplasia, including cervical cancer in its host (rhesus macaques), encodes a functional C-terminal PBM whose integrity is required for transformation of rodent cell lines [44].

But what of the targets of this virally encoded PBM? Seventeen PDZ proteins that interact with the E6 proteins have been identified to date and in the majority of cases (but not all) binding leads to degradation of the PDZ protein *via* the proteasome [107,108] (Table 2). The PDZ substrates are largely involved in defining cell polarity, cell-cell attachment, and organizing cell signalling pathways associated with these functions (Table 2). Notably, components of the three apico-basal polarity complexes, Crumbs, Par and Scribble are E6 targets, suggesting that deregulation of pathways controlled by these complexes is an important function (Figure 1).

The Scribble component DLG1 was the first PDZ protein identified as an E6 binding partner and is targeted for degradation [104,109]. During epithelial differentiation, the localization of DLG1 changes; in the basal proliferating compartment it is cytoplasmic and nuclear but in more differentiated cells present at the cell periphery, indicating location specific functions [3,123]. It is the nuclear phospho-DLG1 forms that are preferentially targets for proteasomal degradation by HPV16 E6 [124,125], suggesting that nuclear DLG1 may encode tumour suppressor activity [3]. But proteasomal degradation of DLG1 is perhaps not the only outcome of E6 binding since several studies have identified functional E6/DLG1 complexes. In HPV16-positive cervical cancer cells, RhoG activity is upregulated and E6 contributes to this upregulation by forming a complex with DLG1 and a RhoG guanine exchange factor (SGEF) [110]. Moreover, the E6-DLG1-SGEF complex contributes to invasive phenotype of the cells, indicating that DLG1 acquires oncogenic functions in the presence of the viral oncoprotein [110], as has been noted with Ad9 E4ORF1 (see section on adenoviruses). In HPV positive cervical cancers, the intercellular communication channels known as gap junctions are lost and E6 has been found to be in complex with DLG1 and the gap junction component connexin protein Cx43 in cervical cancer cells, and this complex may act to inhibit normal trafficking of connexin to the cell membrane [111,126]. Gap junctions and their components, particularly Cx43 play an important role in cell invasion and the E6-DLG1-Cx43 complex may contributes to metastatic properties of cervical cancer cells (Figure 1). Therefore, in HPV infections the tumour suppressor forms of DLG that are involved in the negative regulation of cell proliferation might be the initial target of the E6 PBM, but during disease progression DLG1, either through mislocalization and/or the stabilization of specific pools, acquires oncogenic functions mediated by interaction with E6 [3,127].

**Table 2:** PDZ proteins associated with high-risk alpha HPV E6 proteins^1^.

PDZ Protein (Gene Symbol)	Alternative Name(s)	Cellular Functions	Effect of E6 on PDZ Protein	References
DLG1	SAP97	Cell polarity, proliferation, asymmetric cell division, migration and adhesion	Degradation, complex formation with subcellular pools of DLG1	[104,109,110,111]
SCRIB	Scribble, Vartul, LAP4	Cell polarity, proliferation, apoptotic signalling	Degradation	[112]
MPDZ	MUPP1	Tight junction localization	Degradation	[40]
MAGI-1	AIP3, WWP3	Tight junction integrity, cell growth.	Degradation	[38]
MAGI-2	AIP1	Tight junction function, planar cell polarity, proliferation and motility	Degradation	[113]
MAGI-3		Cell polarity, receptor trafficking, cell survival	Degradation	[113]
TAX1BP3	TIP-1	Cell polarity, cell motility, ion transport	Complex formation	[114]
GIPC1	Synectin, TIP-2	Cell surface receptor expression and trafficking, TGFβ signalling,	Degradation	[115]
INADL	PATJ	Tight junction formation, cell polarity	Degradation	[41,46]
DLG4	PSD95, SAP90, TIP15	Receptor clustering in neuronal cells, epithelial cell polarization	Degradation	[116]
PTPN3	PTPH1	Tyrosine phosphatase, cell growth regulator	Degradation	[103,117]
GOPC	CAL	Intracellular trafficking	Degradation	[118]
PTPN13	FAP-1, PTPL-1, PTP-BAS, PTPBL	Fas-associated phosphatase, cell growth signalling, apoptotic signalling	Degradation	[119]
SLC9A3R1	NHERF1, EBP50	G-protein couple receptor regulation, cytoskeleton anchoring	Degradation of phosphorylated forms	[120]
SDCBP2	Syntenin-2	Phosphoinositol mediated cell signalling, cell division and survival	mRNA downregulation	[43]
PARD3	PAR3	Cell polarity, asymmetrical cell division	Altered subcellular localisation	[121]
PDZRN3	LNX3	E3 ubiquitin ligase, planar cell polarity signalling	Degradation	[122]

^1^ Underlined name used in this review.

The other Scribble component targeted by E6 is SCRIB and this interaction disturbs SCRIB localization at the periphery of epithelial cells and induces loss of tight junction integrity [112]. E6 also targets several other PDZ proteins associated with tight junctions, including MAGI-1, MUPP1, PAR3 and PATJ (Table 1; Figure 1). Recent findings have shown that introduction of a mutant form of MAGI-1 that cannot be targeted by E6 for degradation (as it contains a point mutation in the critical residue in PDZ1 to which E6 binds) into HPV18 positive cervical cancer cells enhances the ability of these cells to form junctional complexes [128]. These studies also identified roles for MAGI-1 in the negative control of cell proliferation and as a regulator of apoptosis, indicating that E6 may target different functional pools of the PDZ protein; selective targeting of these may be relevant at different stages of the virus life cycle and/or disease progression [128].

Protection of HPV infected cells against apoptosis also involves E6 PDZ dependent activation of the PI3K-AKT and NF-κB signalling pathways [120,129]; E6 targets the Na^+^/H^+^ exchange regulatory factor (NHERF1), a PDZ protein known to attenuate PI3K signalling through the negative regulator PTEN (Figure 2). HPV E6 is known to stimulate the key regulator of cellular metabolism mTORC1 leading to activation of cap-dependent translation, and the E6 PBM function has been shown to contribute to the enhancement of translation [130] (Figure 2).

One of the most difficult questions to answer is which of E6 PDZ substrate(s) in the growing list are physiologically relevant targets? In metastatic cervical cancers, the reduction in expression of the polarity proteins DLG1 and SCRIB could be E6 mediated via the proteasome [127,131,132]. Indeed, levels of both proteins are rescued upon silencing E6 expression in HPV-positive cervical cancer cell lines, although the most marked change was noted for MAGI-1, with membrane bound and nuclear pools of the protein restored and re-localization of MAGI-1 to tight junctions [133]. PARD3, an E6 substrate that is not degraded but relocalized from apical domain tight junctions to the nucleus, is restored to the junctions upon E6 silencing in the cancer cells [121]. Somewhat surprisingly therefore, the level of expression and localization of endogenous DLG1 and SCRIB proteins remain unaltered in normal human keratinocytes expressing E6 (E6 alone, or E6 and E7 together, or viral oncoprotein expression driven from intact viral genomes) and there are no changes in the expression or localization of these proteins when the E6 PBM is deleted [96,134,135]. Therefore, it is likely that there is differential regulation of PDZ substrates by E6 at the different stages of HPV pathogenesis. Since the activity of the E6 PBM is regulated by phosphorylation this may be one layer of control. The threonine or serine embedded in the E6 PBM is phosphorylated by protein kinases PKA and AKT and negatively regulates binding to PDZ domains, including those of DLG1 and MAGI-1 [136]. Conversely, E6 PBM phosphorylation confers the ability to interact with a number of 14-3-3 isoforms [137,138]. 14-3-3 proteins have a wide range of cellular functions, regulating key proteins involved in signalling pathways associated with transcription, cell cycle control and apoptosis, although the function of 14-3-3 binding to E6 is unclear at this stage. However, expression of a mutant form of HPV18 E6 that cannot be phosphorylated within the PBM (and therefore cannot bind 14-3-3 proteins), but can bind PDZ substrates enhanced the E6 mediated morphological transformation of human keratinocytes [102]. When this same mutation was embedded in intact HPV18 genomes and transfected into primary keratinocytes, there was a marked increase in hyperproliferation of the epidermal keratinocytes [95]. Thus, modulation of the PKA and/or AKT signalling pathways in HPV infections is likely to impact on E6 function and may be a contributing factor to tumour promotion and progression [95,102].

The mechanism of HPV targeting of PDZ substrates is complicated further by finding that E6*, a spliced isoform of E6 which lacks a PBM, is able to target DLG1, PATJ, MAGI-1, and to a lesser extent SCRIB, for proteasomal degradation [45,46] (Figure 1). Moreover, HPV16 E6 and E7 cooperate to induce the degradation of NHERF1. HPV16 E7 promotes the accumulation of phosphorylated forms of NHERF through activation of cyclin-dependent kinases and the modified forms of the PDZ protein are the preferential degradation targets of the E6 PBM [120]. Thus, multiple cooperative mechanisms exist in HPV infections to control PDZ protein expression and function.

Targeting the various PDZ proteins is not equal amongst the different high-risk HPV types and substrate specificity involves the PBM sequence as well as sequences upstream of the PBM [20,116,120,133,139]. For example, both HPV16 and HPV18 E6 proteins are able to target DLG1 and SCRIB, but exhibit different preferences in binding the PDZ proteins; HPV16 E6 preferentially binds SCRIB over DLG1 and *vice versa* for HPV18 [133,139]. The PBM of these two oncoproteins only differs by a single amino acid (the last amino acid) and, notably, the specificity for DLG1 and SCRIB can be switched within the context of the whole E6 protein by switching the last residue [139]. Whether this has relevance to the carcinogenic strength of the virus (HPV16 is the most prevalent high risk type in cancers) is not clear, but it might indicate that the ability to target at least one protein of the polarity Scribble complex is a key function. Differential targeting of PDZ proteins may also have relevance to the pathogenesis of the virus at different anatomical sites. One such target is PTPN13, a non-receptor tyrosine phosphatase (HPV16 and HPV18 E6 PBM also target another family member PTPN3 [103,117]). HPV16 is associated with over 90% of the HPV positive oropharnygeal tumours (tonsil and base of tongue). In human tonsil keratinocytes, the HPV16 E6 PBM contributes to anchorage independent cell growth and synergizes with the small GTPase RAS for invasion *in vivo*, and these malignant hallmarks were abrogated by reintroduction of PTPN13 [119]. Interestingly, up to 20% of HPV-negative head and neck cancers contain disrupting mutation in the PTPN13 gene. Disruption of PTPN13 regulation of the Ras/RAF/MEK/Erk signalling pathways linked through Ephrin B1 receptor-mediated signalling is likely to be important in both HPV positive and negative head and neck tumours [117,140].

The list of E6 PBM targets is still growing with the use of various types of peptide and proteomic-based screening methods; for example the ubiquitin E3 ligase PDZRN3 was recently identified as a potential novel target of HPV16 E6 PBM in two separate studies [48,49]. The interaction has been confirmed as a degradation target of the HPV16 and HPV18 E6 in a PBM dependent mechanism [122]. The E6 mediated loss of PDZRN3 was linked to activation of STAT5β and this pathway is known to be important in amplification of the viral genomes in the upper layers of the infected epithelia by activating the ATM mediated DNA damage response [141]. Thus, the E6 PBM targeting of PDZRN3 may function synergistically with E7 to activate STAT5 and support viral amplification. Other potential novel targets of the E6 PBM include α and β syntrophins, SNTA1 and SNTB1, whose functions include regulating the activity of the GTPase RAC during tight junction assembly, and sorting nexin 27 (SNX27), important in endosomal recycling pathways [48], but whether these are genuine targets of E6, and the function of these interactions in the HPV life cycle and in HPV driven cancers requires further investigation.

Intriguingly, papillomaviruses may also target PDZ proteins by blocking their transcription. The E6 protein of HPV8, a betapapillomavirus implicated in the development of squamous carcinomas of the skin repressed transcription of the PDZ protein syntenin-2, and HPV16 E6 was also able to repress syntenin-2 transcription but to a much lesser extent than the HPV8 protein [43]. Loss of syntenin-2 expression was shown to inhibit differentiation and promote proliferation of human keratinocytes; possibly by deregulation of PIP2 mediated nuclear signalling, a signal transduction pathway linked to epithelial cell growth and differentiation [43,142]. It will be important to establish if other PDZ proteins are similarly deregulated at a transcriptional level in HPV infected and transformed cells.

## 3. Concluding Remarks

One of the striking features of this group of viruses is the commonality in PDZ targets (Figure 1). Many are components of the polarity complexes and the deregulation of the cellular pathways they control is involved in virus infection, transmission, replication, survival and persistence in the host, and formation of new progeny. For many of the viruses, these interactions also play a part in their oncogenic actions and indicates that the deregulation of the PDZ protein controlled pathways is a finely balanced process in the virus life cycle and once this balance is perturbed this can lead, or contribute to oncogenesis. This perhaps is not so surprising when it is known that many of the components of the polarity complexes behave as tumour suppressors in different systems, although this behaviour can be context dependent. The viruses deregulate the PDZ proteins in many different ways—inducing cellular mislocalization, proteasomal degradation, or by binding to the protein to compete with host cell partners, and intriguingly, some viral—PDZ protein associations lead to the PDZ protein acquiring oncogenic functions. Interestingly, many of the virus—PDZ associations discussed here affect the PI3K/AKT signalling pathway that is fundamental to cell growth and survival (Figure 2) and is thus an obvious target for the viruses to exploit for replication. Indeed, many other viruses that are not oncogenic use an array of strategies to interfere with this cell signalling pathway and perhaps some of these may be relevant to the viruses discussed in this review [143]. The use of high-throughput proteomics and construction of large libraries to screen protein-protein interactions is leading to the identification of further PDZ targets for these viruses. Thus, one area to tackle is to confirm those interactions which are physiologically relevant, a problem particularly significant to the high risk HPV E6 proteins which already has a list of seventeen targets and is growing with addition of new potential targets from the large screening arrays; the use of HPV life cycle models will be an important asset in these investigations. It is important that we understand the intricacies of these interaction; when and where they occur during the life cycle and what interactions are involved in oncogenesis, because these interactions are attractive therapeutic targets that can be targeted using disrupting molecules [144,145].
